# Patient motivators to use opioids for acute pain after emergency care

**DOI:** 10.3389/fpain.2023.1151704

**Published:** 2023-09-25

**Authors:** Brittany E. Punches, Jennifer L. Brown, Natalie K. Taul, Hawa A. Sall, Tamilyn Bakas, Gordon L. Gillespie, Jill E. Martin-Boone, Edward W. Boyer, Michael S. Lyons

**Affiliations:** ^1^ College of Nursing, The Ohio State University, Columbus, OH, United States; ^2^ Department of Emergency Medicine, The Ohio State University College of Medicine, Columbus, OH, United States; ^3^ Department of Psychological Sciences, Purdue University, West Lafayette, IN, United States; ^4^ College of Nursing, University of Cincinnati, Cincinnati, OH, United States; ^5^ College of Pharmacy, University of Cincinnati, Cincinnati, OH, United States

**Keywords:** opioid, acute pain, decision-making, emergency care, patient preferences

## Abstract

**Introduction:**

Patients are stakeholders in their own pain management. Factors motivating individuals to seek or use opioids therapeutically for treatment of acute pain are not well characterized but could be targeted to reduce incident iatrogenic opioid use disorder (OUD). Emergency departments (EDs) commonly encounter patients in acute pain for whom decisions regarding opioid therapy are required. Decision-making is necessarily challenged in episodic, unscheduled care settings given time pressure, limited information, and lack of pre-existing patient provider relationship. Patients may decline to take prescribed opioids or conversely seek opioids from other providers or non-medical sources.

**Methods:**

Using a framework analysis approach, we qualitatively analyzed transcripts from 29 patients after discharge from an ED visit for acute pain at a large, urban, academic hospital in the midwestern United States to describe motivating factors influencing patient decisions regarding opioid use for acute pain. A semi-structured interview guide framed participant discussion in either a focus group or interview transcribed and analyzed with conventional content analysis.

**Results:**

Four major themes emerged from our analysis including a) pain management literacy, b) control preferences, c) risk tolerance, and d) cues to action.

**Discussion:**

Our findings suggest targets for future intervention development and a framework to guide the engagement of patients as stakeholders in their own acute pain management.

## 1. Introduction

Opioid use disorder (OUD) incurs tremendous individual and societal costs (1–7). Healthcare costs associated with response to overdose emergencies as well as injuries and complications of OUD are also exorbitant (8). Increasing evidence indicates the development of OUD is frequently iatrogenic at least in part, whereby OUD is could be a consequence of opioid use that began as therapeutic (9–15).

This evidence surrounding opioid use and various policy initiatives have led to significant reductions in opioid prescribing with uncertain effects (9, 16–19). Presumably, there are still patients who receive therapeutic opioids that might be avoided, patients for whom therapeutic opioids cannot be avoided, and others denied opioids unnecessarily (20). Amidst ambiguity in balancing individualized OUD risk versus pain severity, shared-decision making in which patients are primary stakeholders in pain management decisions is likely to continue. At a minimum, patients are primary agents in the decision to seek or use opioids non-medically when pain is unmanaged through medical sources (20, 21). Emergency medicine prescribers are especially challenged. Patient-provider interactions in the emergency department (ED) are necessarily brief, often with limited information and no pre-existing or longitudinal patient relationship, all presenting barriers to risk assessment and shared decision-making (20, 22). Moreover, the risk of short-term, low-potency opioid therapy is even less well characterized than is opioid therapy for chronic pain management (10, 11, 23, 24).

Patients are stakeholders in their own pain management. Not only may they influence provider prescribing decisions, but they may also decline to take prescribed opioids or conversely seek opioids from other providers or non-medical sources. It follows that patients’ individual motivations to take or avoid opioids could be targeted for development of future interventions to reduce OUD or improve pain management. We sought to qualitatively describe motivating factors influencing ED patient decisions for opioid use when presenting for care in an acute pain.

## 2. Materials and methods

### 2.1. Design

This study is grounded in theoretical underpinnings of the Health Belief Model (25) and followed COREQ standards for qualitative research (26). A framework analysis method of qualitative analysis was used to explore motivating factors that influence patient decisions to use opioids after emergency care (27). This method was chosen for its ability to allow the data to emerge directly from the words of the participants but also place minimal interpretation on their thoughts. The study was approved by the local Institutional Review Board.

### 2.2. Setting and sample

Purposive sampling was used to recruit patients between August and December 2021 from an urban, academic, adult trauma center emergency department with approximately 75,000 visits annually.

Potential participants were screened for preliminary eligibility via the electronic health record during the ED visit, and were subsequently consented and enrolled by trained study staff. Participants were required to be at least 18 years of age, English-speaking, and having an ED visit related to acute pain (i.e., due to recent illness or injury). Participants were excluded if they were prisoners/in custody, previously enrolled, suicidal, lacked capacity to consent and participate, or pregnant/trying to conceive, but were not excluded for pre-existing opioid use disorder, chronic pain, or chronic opioid use. Participants completed baseline surveys and were scheduled for follow-up interview/focus group at approximately 7 days (±4 days) after ED discharge. Study staff scheduled focus groups (*n* = 5) with 2–3 participants based on similar histories of opioid exposure (e.g., opioid naïve, sporadic use, and consistent use) since prior exposure is intuitively associated with current perceptions of opioids. Individuals with similar histories of opioid use were brought together in focus groups. When an eligible participants prior opioid history was not consistent with a pending focus group, they were scheduled for individual interviews.

Participants completed a baseline questionnaire assessing sociodemographic characteristics, basic health information, and current substance use based on Diagnostic and Statistical Manual of Mental Disorders, 5th revision (DSM-5) criteria (28). All baseline data were collected and managed in a secure Research Electronic Data Capture (REDCap) system database (29). Participants received a $20 gift card for completing the survey in the ED.

### 2.3. Qualitative assessment

We used a semi-structured interview guide, informed by the Health Belief Model and previous literature (20) to assure that all participants received the same core questions during the interview. Interviews were conducted by two members of the study team, one of whom is a licensed social worker (NT) and the other a PhD prepared emergency nurse (BP). Conversations occurred via Microsoft Teams and were digitally recorded and subsequently transcribed and verified for accuracy. Focus group discussion lasted between 42 and 64 min and individual interviews ranged from 25 to 76 min. Although full study consent was obtained in the ED, the qualitative follow-up visit began with reiteration of study purpose and verbal consent for recording. In order to avoid stigma and response bias, question prompts asked participants to reflect on peer behaviors in the community generally, allowing participants to avoid self-report of their own behaviors. However, we allowed participants to describe their own thoughts, perceptions, and behaviors if desired. Participants received a $25 gift card for completing a focus group or interview.

### 2.4. Data analysis

Sample characteristics were descriptively analyzed. Using the framework analysis approach, two members of the research team coded categories with a predetermined code list (30, 31), allowing new concepts to emerge from the voice of the participants. In phase 1 (familiarization), the analysis team worked first independently and then together using an iterative content analysis approach (31), eventually achieving consensus in regard to important themes and ideas. Next, the team met to develop our thematic hierarchy (Figure 2). In phase 3 of analysis (indexing), the qualitative data were coded according to the developed thematic framework. During phase 4 (charting), we compiled direct quotes from the participants with their corresponding headings and definitions. Finally, in phase 5 (mapping and interpretation), the analysis team established supporting literature of the concepts in our thematic framework. We enrolled participants until we achieved redundancy in content, achieving thematic saturation (32). Any disagreements were brought to the other members of the research team and discussed until consensus was achieved. Credibility was upheld by minimizing investigator bias through adhering to inclusion criteria and maintaining an audit trail. Reliability was accounted for through standardized procedures, and confirmability was supported by triangulation of investigators and audit trail (32). The qualitative software NVIVO (QSR International, Burlington, MA) was used for the data analysis procedures.

## 3. Results

**Figure 1:**
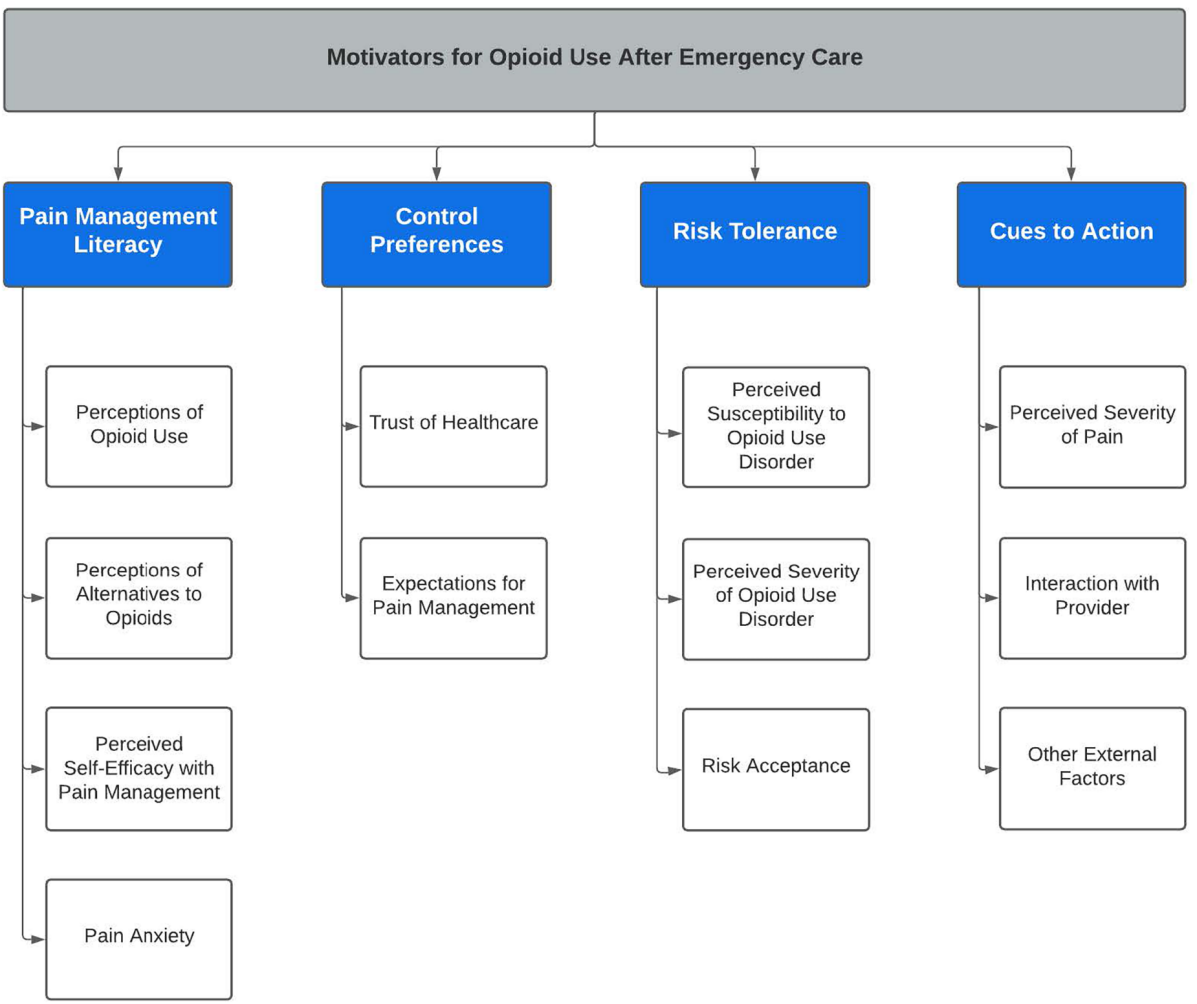
Thematic Hierarchy of Patient Motivators to use Opioids.

We approached 217 patients for eligibility, of which 62 (28.6%) were eligible and consented to procedures. Of the 29 participants who completed either a focus group (*n* = 11) or interview (*n* = 18), the majority were white (16, 55.1%) and male (19, 65.5%). Seven (24.1%) of the sample had never used opioids, and four (13.8%) had current OUD (Table 1). Four major themes emerged from the analysis (Figure 1), which represent motivating factors influencing patient decisions to use opioids for pain management. These themes include (a) pain management literacy, (b) control preferences, (c) risk tolerance, and (d) cues to action. There were no differences in themes across varying histories of opioid use.

**Table 1:** Characteristics of study population.

	*N* = 29	(%)
Age—years, mean (range)	41	(20–79)
Race/Ethnicity		
White, Non-Hispanic	16	(55.1)
Black/African American	12	(41.4)
Black, Hispanic	1	(3.4)
Gender		
Male	19	(65.5)
Female	10	(34.5)
Marital Status		
Married/Relationship	12	(41.4)
Divorced/Separated	3	(10.3)
Never Married/Not reported	14	(48.3)
Insurance		
Government supported	17	(58.6)
Private	12	(41.4)
Employment Status		
Full-time/Part-time	16	(55.2)
Not employed/student	13	(44.8)
History of Chronic Pain		
Never	15	(51.7)
Yes, Currently	12	(41.4)
Yes, Not Current	2	(6.9)
History of Opioid Use		
Never	7	(24.1)
Current/Last 7 day opioid use	5	(17.2)
Sporadic past opioid use	13	(44.8)
Current OUD/In treatment	4	(13.8)

### 3.1. Theme 1: pain management literacy

All participants described aspects of knowledge, attitudes, and beliefs about pain management including using opioids for pain and alternative therapies for pain management. Grounded in the Health Belief Model theoretical constructs of perceived susceptibility, severity, benefits, and barriers (25), we found that an individual’s pain management literacy combines their personal experiences and information gained from providers, family, and peers. There was also mention of other aspects of knowledge such as internet sources and schooling. Finally, perceptions of pain included pain management self-efficacy and pain anxieties for the ability to apply knowledge to manage their own pain.

#### 3.1.1. Sub-theme: perceptions of opioid use

Participants frequently discussed beliefs and knowledge of opioids including their beliefs on why individuals would or would not take opioids for pain. This included benefits of opioids such as relieving pain, euphoric/pleasant feelings from the opioids, as well as being able to escape problems. Some stated sentiments of not being able to be in pain due to their commitments as a caregiver or needing to be able to work.

One participant stated, “*(Opioid medication) helps. You know it doesn't take it totally away, but it makes it a point to where I can do everything that I need to do… You know to take care of my daughters effectively. Play with them, you know, to just have a normal life*” (45 y/o black female).

There were also unique perspectives of use, “*people tell me their sex drive, (that) they can last longer (when they take opioids). So some people take it just for that. Some people don't even take it for the pain*” (40 y/o black male).

Reasons that would prevent opioid use for pain management included potential side effects such as nausea, grogginess, and not being able to think clearly. A participant described her side effects with opioids, “*took one and then of course by that time I was getting nauseous. ‘cause that’s what they do, to me they make me sick at my stomach… Like I've had after surgery, I'll throw up. So they're not good for me to take anyway”* (79 y/o white female).

Additionally, some brought up thoughts that some people may not take their opioid prescription medications due to the potential for diversion of opioids for financial reasons: “*just getting pills just to sell them, and they might not necessarily take them but they know how to get money off them. I definitely say they’d do it”* (53 y/o black male).

#### 3.1.2. Sub-theme: access, awareness, and acceptance of alternatives to opioids

Individuals in the focus groups and interviews expressed varying knowledge and beliefs about alternatives to opioids. Participants generally expressed a lack of knowledge of and access to alternative therapies to opioids including confusion on types of pain medications that were non-opioids and available over the counter. When discussing awareness of alternative therapies for pain management, individuals mentioned learning from family members as well as a lack of knowledge about pain management outside of healthcare. Finally, individuals expressed sentiments that alternatives to opioids either do not work or take too long to work and requiring an immediate solution to the pain. The majority of the responses indicated financial factors playing into reasons for their use of alternatives to opioids.

##### 3.1.2.1. Access

*“And it tends to be with people who had just have more access (to alternatives to opioids), more money, and can afford it”* (31 y/o white male).

##### 3.1.2.2. Awareness

“*I mean, I'm just being honest. In a daily family like, in a low (income) poverty family, they're not going to talk about something like (alternative methods for pain). Maybe in an upper-class neighborhood, but no, not in a low (income) poverty neighborhood, no*” (47 y/o black female).

##### 3.1.2.3. Acceptance

*“At times (opioids are) the cheapest available…Like right now I'm struggling with this pain. I need to be at work (and) I don't have enough time to pursue my own healing and so you end up maybe giving into a pill that you can pop and feel better. A few hours pop another pill, as opposed to maybe getting sessions of physical therapy and extensive imaging to find out what’s really is the problem and therefore manage it appropriately*” (53 y/o black male).

#### 3.1.3. Sub-theme: perceived self-efficacy with pain management

In this subtheme, participants describe confidence and beliefs about their abilities to manage pain with or without opioids. This subtheme combines their application of knowledge and beliefs of opioids and alternatives to opioids in pain management. Some participants also noted generational acceptance of pain, “*We get soft and we're not willing to deal with some pain, and like I can deal with some*” (78 y/o White male), and a need for immediate pain relief, “*we’re living in a microwave world so much that ‘I don't care. I just want this pain gone’*” (28 y/o black male). Finally, participants described personal abilities to manage pain due to previous pain experiences:

*“I'm not a big med taker in general, so I can endure some pain. I mean, as a side note, I have (…), so I am pretty much constantly in some type of discomfort. I am on medication for it, but it doesn't work”* (51 y/o white female).

#### 3.1.4. Sub-theme: pain anxiety

This refers to a person’s subjective perception of chances of and reactions to pain. This may include a fear of pain, “*When it comes to (pain) I'm a baby, I have to get something for it*” (27 y/o white female). Additionally, we found that some people confused the concept of pain and physiologic drug tolerance, stating they believed they had a high pain tolerance and therefore required very strong opioid medications:

“*I have a very high tolerance for pain… they wanted me to take ibuprofen where ibuprofen don't do anything for me …They gave me hydrocodone and they didn't do anything …So I guess my pain level is more intense to other people to where I really need something really strong*” (57 y/o black female).

### 3.2. Theme 2: control preferences

This domain describes individuals’ beliefs and trust surrounding healthcare as well as specific expectations surrounding pain management. Subthemes included in this domain are trust of health care and expectations for pain management. We found that individuals were explicit whether they were going to follow provider instructions versus display a distrust and skepticism for aligning with provider recommendations.

#### 3.2.1. Sub-theme: trust of health care

The theme of trust in healthcare describes both the confidence and reliance in their healthcare provider’s decisions surrounding pain management and opioid use for the individual patient. Within this theme, participants described power dynamics and willingness to put faith in the medical provider’s decision as well as personal philosophies to approaching healthcare and medications. Participants described instances where they just went along with what the healthcare provider said, following instructions:

*“I felt like I honestly at that point when they did the hydromorphone, I didn't really have an option or an ability to say, hey, let’s try something lower because I was feeling pain from my hand… I think I was trusting the situation more than advocating for myself”* (51 y/o white female).

Additionally, some participants described skepticism and lack of trust related to the healthcare system:

*“Just there’s a lack of trust. I think, uh, going on with medical community in general right now … and so it’s sort of seen as, a money, money and a prize and not actually healthcare enterprise” (31 y/o white male)*.

Finally, some participants stated that they didn’t like taking medication because it went against their personal beliefs:

*“I'm not gonna take a bunch of medicine that makes me painless, because pain in our body is an alarm system that says there’s something wrong… I wouldn't take a pain medicine if I thought it was just gonna mask my problem and the problem wasn't going to go away”* (78 y/o white male).

#### 3.2.2. Sub-theme: expectations for pain management

The theme of expectations for pain management portrays beliefs and ideologies when approaching pain management. Within this theme, participants describe whether a diagnosis and pain were validated by the healthcare provider. Also embedded in this theme, participants stated that receiving a prescription pain medication that was not available over the counter was a method of validation of their individual concerns surrounding pain, “*'cause they feel like the opioid is stronger and it’s gonna work better than like that regular medicine*” (32 y/o black male). Another participant described the lack of validation of her symptoms, “*I'm in terrific pain… for her not to give me any pain medicine … she didn’t take a x-ray or anything … I'm ready for a second opinion”*. One participant discussed dissatisfaction when receiving an opioid prescription against his wishes:

*“they prescribed an opioid. And when I specifically said I didn't want that, they still did not give me a different prescription or even want to have a conversation… but that still left a really bad taste in my mouth and especially because I made them aware of the reasons why I didn't want that prescription” (22 y/o white male)*.

### 3.3. Theme 3: risk tolerance

Participants describe aspects of risk tolerance including subthemes of risk acceptance, perceived severity of OUD, and perceived susceptibility to OUD. One participant said: “*But I do not want to deal (with) the opioid at all. I was prescribed that one time and I felt… I didn’t feel comfortable…I mean the pain was subsided, but I was not feeling confident taking it, with a chance of me getting too addicted to it and everything else*” (53 y/o black male). Notably, most of the participants agreed that opioid use disorder was a serious disease and many stated they knew of individuals who transitioned from opioid prescriptions to injection drug use.

#### 3.3.1. Sub-theme: risk acceptance

This is when a participant describes being knowledgeable about the risks of opioid use and would choose to take an opioid for pain despite the known risks. This would include aspects of not caring about the risk, just wanting the pain gone, or believing that there would be a way to prevent or treat opioid use disorder. One participant stated, “*if I'm in like 9 out of 10 out of pain and …a doctor prescribes me a … strong medicine that … could be addictive … I might take that risk just to get rid of that pain. … you're obviously going to take that step or take that risk*“ (20 y/o white male). Another participant response described some of the thoughts that are considered when weighing benefits and risks of an opioid:

*“Some people actually like the feeling of being high off the pain medicines or just feeling real relaxed. And they ain't gotta deal with the pain. And then you got some people that it’s like, ‘alright, I will on … how just pain medicine and you got me feeling good little bit from my pain’. But it’s like I don't wanna be like this all the time ‘cause now you interfering with other things in your life. It ain't just dealing with that right now, you interfering with other things. You might be too sleepy and miss something or you might be too drowsy to do something”* (32 y/o black male).

#### 3.3.2. Sub-theme: perceived severity of opioid use disorder (OUD)

This refers to a person’s feelings on the seriousness of developing OUD. The theme of perceived severity of OUD can include personal beliefs about OUD and the complications it has on individuals’ lives, violence and crime surrounding OUD, and their specific fears of developing OUD. An individual discussing both fear of addiction and awareness of severity stated:

“*A lot of them just might not want to take (opioid prescription) because they see other people struggling. You know with (addiction), and they're like… I don't wanna do that to myself. I see these people out here, homeless, struggling, bumming for money. You know, don't know when they're going to get their next fix. So they're like hell I might as well throw them (pills) away because I don't want to be like that person”* (27 y/o white female).

#### 3.3.3. Sub-theme: perceived susceptibility to opioid use disorder

This refers to a person’s subjective perception of the risk of acquiring OUD. This theme combines traits and beliefs participants describe as affecting an individual’s vulnerability to OUD. Characteristics believed to increase susceptibility include personality, genetics/family history, friends, not having a ’strong mind’, not taking opioids as medically prescribed, and varying other beliefs such as ‘it won’t be me’. One participant stated, “*a lot of people have pride to the extent that they think well other people, but not me*” (25 y/o white male). Another person indicating that she was not at risk stated: “*I won't worry about getting addicted because I know how strong my mind is”* (57 y/o black female).

### 3.4. Theme 4: cues to action

This final theme describes an internal or external stimulus that can trigger the decision-making process in order to take an opioid for pain. Specific cues to action include subthemes of (1) perceived severity of pain, (2) interactions with providers, and (3) other external factors that influence their decision.

#### 3.4.1. Sub-theme: perceived severity of pain

In this subtheme, participants detail components of pain severity that may motivate them to take an opioid for pain relief. During the discussions, aspects of interferences with sleep, daily activities, and work were prominent. Additionally, discussions of physical responses to pain such as crying or screaming were mentioned when relating the severity of pain to motivation to take an opioid. Several participants discussed that high levels of pain would motivate an individual to take a medication even if they initially did not intend to, as one participant stated, “*there’s only so much pain your body can take and that’s why they have torture chambers to make you talk”* (78 y/o white male). Another agreed, “*people, if they're in enough pain, will cave to it, which can obviously create a pretty slippery slope”* (25 y/o white male).

#### 3.4.2. Sub-theme: interaction with provider

Participants reported mixed reactions when discussing pain management with their emergency care provider and their choices to use opioid after discharge. Of the participants interviewed, 31% (*n* = 9) stated that emergency care providers are prescribing fewer opioids now due to the opioid crisis and legislative changes. One participant stated:

“*But if the pending on what type of pain there in… they may get a Vicodin or Percocet, but they're not gonna get a big quantity of it. ‘cause I mean, with the laws nowadays they don't prescribe pain meds like that anymore”* (47 black female).

Notably, many participants thought that emergency care providers would be more likely to give opioid medications for pain management due to the hurried nature of emergency care: “*because they wanna get them out (of) the emergency room. Quick, fast and in a hurry, so that’s a quick fix*” (47 y/o black female) and with little information, “*a lot of time (there) are options to find out what’s exactly wrong, and so just easier to treat the pain than it is necessary to treat whatever is underlying it. Not having a complete medical record*” (31 y/o white male).

#### 3.4.3. Sub-theme: other external factors

Participants also described other external motivators that cued them to take or not take an opioid for pain management. These external factors included friends and family members, “*I had refused to (take the opioid), it took a lot of convincing from family as well as my doctors to do something, because it had came to the point to where I was in so much pain*” (45 y/o black female). Additional external factors include stigma, societal acceptability, and fear of being labeled as a drug user, “*I do believe that pain medication now has a stigma, because of everything you hear on social media, the news and any kind of reporting*” (35 y/o white female).

## 4. Discussion

This study identified discrete categories of information, perceptions, and ultimately motivations that influence patient decisions to use opioids. Our qualitative analysis found four categories of patient motivators to use opioids including: pain management literacy, control preferences, risk tolerance, and cues to action. These results suggest potential targets to engage the patient as a stakeholder in their own acute pain management, either reducing or increasing opioid use depending on the desired outcome. Knowing key patient motivators for decisions to use opioids for acute pain after an emergency care visit may be foundational to the development of personalized, patient-centered interventions for acute pain after an emergency department (ED) visit.

The broad concept of health literacy includes the ability to understand, interpret, and apply health information and services to make informed decisions (33). Our findings on the importance of pain management literacy in patient decision-making aligns with other studies in which individuals with lower health literacy were more likely to misuse opioids, have higher levels of pain severity, and have increased catastrophizing (34, 35). Participants in our study had many misconceptions and knowledge gaps about pain, opioids, and alternatives to opioids. Preferences for uptake of pain management literacy included generational transfer of knowledge of pain management strategies andability to access health and pain management information online.

Control preferences refers to patient preferences for the degree of control in healthcare decision making. Participants vocalized preferences for control in pain management, combining their trust in healthcare as well as expectations for pain management as key to whether or not they would adhere to recommendations or seek opioids from alternative sources. Several participants varied in the source of their discontent in pain management, with some even upset they received opioids when they did not wish them. Our findings are consistent with previous studies describing discontent with pain management as a trigger to seek opioids from non-medical sources (20), as well as studies linking control preferences to clinical outcomes and satisfaction with care (36–38).

Participants were aware of potential risks of opioid use, though some focused on factors such as personality traits and family history rather than the opioids themselves. Having a “strong mind” and taking opioids as medically intended was thought to be protective. This finding is consistent with beliefs that OUD is a moral failing and stigmas against individuals who have developed OUD as having a “weaker mind” (39, 40). It was generally unclear how individuals translated their views of the role of risk factors into their own personal risk perception. This raises the critical importance of research to scientifically quantify individualized risk. It will not be useful to emphasize overall risks or safety of opioids in counseling sessions if individuals are prone to viewing their own risk as different from the general population.

Cues to action are considered either internal or external influences to trigger a decision to accept an action, such as taking an opioid medication (41). Participants stated that interactions with providers and advice from others such as family members influenced their actions and decisions to take opioids for pain. This finding is consistent with other literature where patient experience and interactions with providers have implications for healthcare outcomes beyond the ED encounter (20, 42–45). Moreover, it is important to consider that external influences beyond the ED encounter may influence opioid use for acute pain management. Risk counseling and shared decision-making strategies are important in opioid use mitigation; however, other influences outside of the provider’s control may trigger later opioid use.

Our findings should be considered in context with several limitations. We did achieve thematic saturation using a semi-structured interview guide, but qualitative research is an in-depth analysis of a sample that may not be generalizable to the larger population. The description of our setting and sample characteristics should inform transferability. Similarly, we are not able quantify prevalence of these patient-reported cognitive factors or to investigate strength of associations or mediating effects. Our findings may have been influenced by our thematic framework guided by the Health Belief Model and previous literature. Finally, while this study was focused on the patient perspective of opioid use, the authors note that external influences such as provider training, system-level factors, and societal views of pain care may inform patient experiences.

## 5. Conclusion

Our study provides a framework for understanding patient motivations to take or avoid opioids. This warrants future investigation to determine the prevalence of these factors, their associations and predictive capacity, and the degree to which these cognitive factors and behaviors are modifiable. This demonstrates potential targets for the development of future interventions to reduce OUD or improve pain management after emergency care.

## Data Availability

The raw data supporting the conclusions of this article will be made available by the authors, without undue reservation.
