# Supplementary material for: Patient motivators to use opioids for acute pain after emergency care
Source: Front Pain Res (Lausanne). 2023 Sep 25;4:1151704. doi: 10.3389/fpain.2023.1151704 (PMC10560756; doi:10.3389/fpain.2023.1151704)
Supplement: Supplementary file 1 [file Datasheet1.docx]

**Appendix A: Interview Guide for Interview or Focus Group Questions.**

**Introduction**

Thank you all for coming today. My name is [insert name]. We are here today to talk about what **patients in your community** think about opioids and pain. **We are *NOT* specifically asking about your individual views or practices, although we understand if you choose to share your personal experiences to help explain your perspective.** Your help is much appreciated!

Before we get started, I’d like to review several important rules and reminders:

1. You do not need to respond to any questions you don't feel comfortable answering.
2. There are no right or wrong answers.
3. You should feel free to disagree with what other people say but do so respectfully. Everyone’s opinions and views are valuable.
4. The session is being audio recorded and then transcribed into text so that we can refer back to our discussion and not miss what you say. However, your full name will not be connected to the recording that is being made and we will destroy the recording when the project is complete.

For this reason, we ask that you don’t use specific names when discussing topics. This discussion is all confidential and this will not be shared with your doctor or be put in your medical record. We’ll also be taking notes during the session. This is to help us remember what you say today.

1. What is said here should stay here. Please respect the privacy of group members by not repeating what is said today outside of the group.
2. Each person will have the opportunity to respond to the question but we ask that one person speaks at a time

***Note to interviewer:*** *Go around the room and attempt to elicit responses from each individual. Interactive discussion should occur but try to allow each individual to respond to each question.*

**Reasons why patients go to the Emergency Department:**

1. Tell me all of the reasons people in this community may choose to visit the emergency department.
2. Are these reasons different from why someone may or may not go to their usual (primary) doctor?

**Reasons why patients receive pain medicine in the ED.**

1. Many of the responses sounded painful. Tell me all of the reasons you think people in this community may receive pain medicine in the emergency department/hospital.

PROBES: What types of pain medicines do you think people get in the emergency department? Why do you think some people get over the counter (e.g., ibuprofen/Advil/Tylenol) versus prescription (e.g., opioids, morphine)?

**Reasons why patients receive prescription pain medicine**

1. Tell me all of the reasons why people in this community may receive a prescription for pain medicines when they leave the emergency department/hospital.
2. Tell me all of the reasons why people in this community may not receive a prescription for opioid pain medicines when they are discharged.

PROBES: What types of prescriptions for pain medicines do you think people receive? Why do some people get over the counter (e.g., ibuprofen/Advil/Tylenol) versus prescription (e.g., opioids, morphine)?

**Reasons for taking opioid pain medicine.**

1. Tell me all the reasons why a person in this community **may take an opioid pain** medicine after their emergency room visit.
2. Are there differences in whether the opioid prescription was given by emergency doctor or primary doctor?
3. Tell me all of the reasons why a person **may not take an opioid** pain medicine that was prescribed.
4. Tell me about some side effects that might prevent someone from taking an opioid prescription.
5. What concerns (if any) do people in your community have about the possible addictive potential of opioids when taking for pain?
6. Do people in your community think that addiction/ opioid use disorder is serious?
7. Do people in your community think that they are at risk for addiction?

PROBE: family history? Friends? Pain? Mental health?

**Reasons for opioid misuse/OUD/addiction**

1. Tell me all of the reasons why a person in this community might start taking opioids not as prescribed.

PROBE: Overtake or undertake? More/Less frequently or More/less in quantity?

1. Tell me all of the reasons why someone might get opioids other than from a prescription from their physician/prescriber.
2. Tell me all of the reasons why someone might take opioids even though they are at risk for being addicted/getting hooked.
3. How do you manage pain? (Chronic pain)
4. Closing remarks: Is there anything final that you may want to add?
